# Sixty years of work on Italy’s Orthopteroids biodiversity, the big data of Galvagni collection

**DOI:** 10.3897/BDJ.9.e65953

**Published:** 2021-06-30

**Authors:** Filippo Maria Maria Buzzetti, Gionata Stancher, Federico Marangoni

**Affiliations:** 1 Fondazione Museo Civico di Rovereto, Rovereto, Italy Fondazione Museo Civico di Rovereto Rovereto Italy

**Keywords:** Natural Science Museum, Italy, entomology collection, biodiversity

## Abstract

**Background:**

Historical natural history collections are very important for the study of nature and environmental protection of the environment, these being the depository of essential information. The Fondazione Museo Civico di Rovereto holds two major Orthopteroid insect collections that make this Museum a landmark on Italian and Mediterranean Orthoptera diversity. Databasing the Galvagni Collection allows considerations on geographic and taxonomic coverage by specialist researchers.

**New information:**

Databasing of the Galvagni Collection makes possible considerations on the late specialist research, geographic and taxonomic coverage.

## Introduction

The Fondazione Museo Civico of Rovereto (FMCR) is an Italian civic museum founded in 1851. The Museum contains many collections ranging from natural sciences and archaeology to art, but the entomological, botanical and archaeological collections are of greater relevance. In fact, these count more than 286,980 exhibits and are the data source of many scientific publications. The first collections date back to the years of the Museum foundation, but unfortunately, part of these were lost during World War I. The collections of the FMCR have grown during nearly 200 years of the foundation , so that the number of collections and exhibits, curated in the Museum, is continuously increasing. Currently, at the FMCR, there are four entomological collections of both national and international relevance given the presence of many types: the Bernardino Halbherr Collection is composed mainly of Coleoptera, the Livio Tamanini Collection consists of Hemiptera and Coleoptera, the Antonio Galvagni Collection gathers Orthopteroid Insects and the Collection, recently donated by Paolo Fontana, also about Orthopteroid Insects. The first three Collections are mainly composed of specimens collected within the Italian borders, while, on the other hand, specimens of the Fontana Collection come from all over the world, but mainly Italy and Central America. Museum collections are reservoirs of non-renewable information (Winston 2007), so digitisation work is needed to prevent such information from being lost or destroyed due to external events or mismanagement (Andreone et al. 2014). The aim of digitisation is to make the access and study of this information easier for experts and amateurs who want to compare their specimens with the large reference collections that carry out the task of archiving nature (Hoeksema et al. 2011, Smith and Blagoderov 2012). Noteworthy is the Galvagni Collection that covers 60 years of sampling in all regions of Italy and with an excellent representation of the species present in the territory. All this material is an irreplaceable resource and an excellent starting point for carrying out studies on ecology and the variation of biodiversity over time, particularly during the current period of anthropological change (Chapman 2005, Hill et al. 2012, Hoeksema et al. 2011, Alessandri et al. 2019). The purpose of this focus on the Galvagni Collection is to enhance this donation acquired by FMCR in 2015, communicating to experts and amateurs the considerable amount of useful data held by the preserved specimens. Antonio Galvagni (24 May 1924 - 30 April 2015) was a key entomologist on Italian Orthoptera and related orders of Insects (Massa and Fontana 2016). The Galvagni Collection consists of a systematic part and a miscellaneous part for a total of 350 entomological standard boxes, containing 382 types of which 30 are holotypes. The number of types is likely to increase over time, for example, thanks to the help of six specimens of this Collection, a new species of grasshopper has been described for the Italian territory (Fontana et al. 2019).

## General description

### Additional information

**Figure 1.**
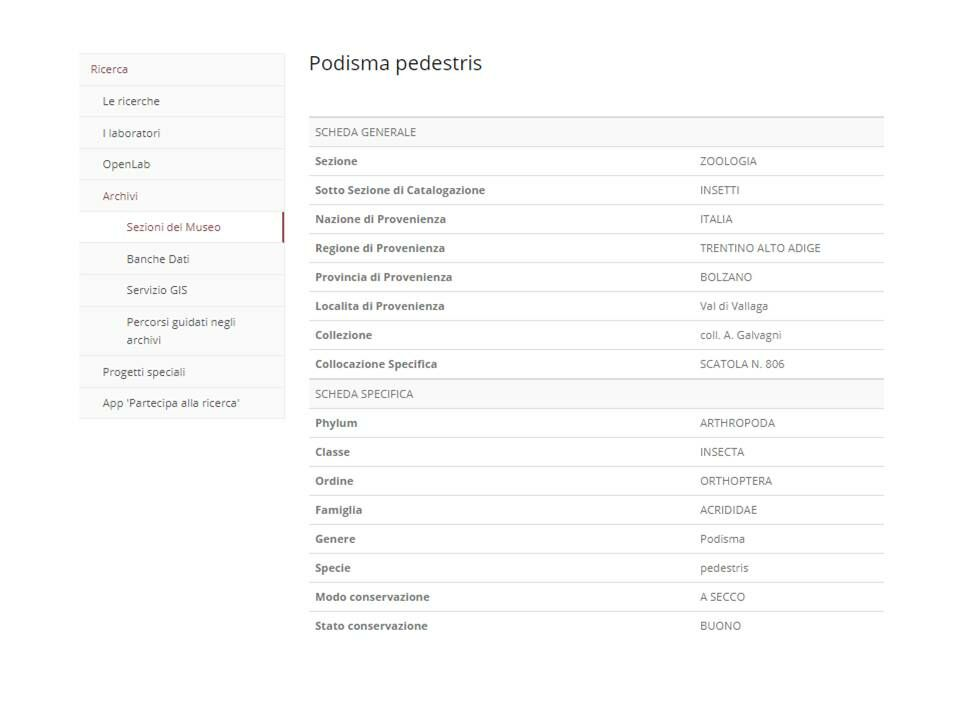
Database of FMCR available on www.fondazionemcr.it subsection Archives.

The work carried out on the Galvagni Collection took three years of work (2016 - 2019) between reorganisation, restoration of some boxes and digital databasing. The collection as it entered the Museum was in a good state, even after some years of no maintenance by the owner. Nevertheless, to avoid any sort of possible infestation, it was subjected to freezing treatment using large refrigerators present in the Museum. Afterwards, the entomological boxes in the FMCR deposit began to be arranged, cleaned and restored. Finally the systematic collection, that part of the Galvagni Collection identified and arranged according to current taxonomy, was digitally databased in the Museum catalogue. This consists of 219 boxes and is available on the website www.fondazionemcr.it in the subsection Archives/Sections of the Museum/Zoology-Insects, after registration in the Museum portal (Fig. 1) or is downloadable here as a supplementary file (Suppl. material 1). The systematic collection contains samples pinned or glued on a label for a total of 32,046 specimens and some extracts of genitals or glands prepared on a slide, mostly in excellent condition. All specimens are accompanied by an identification label and collecting locality label indicating country, region, province, municipality, location, altitude and date of collection. On some specimens, not collected directly by Antonio Galvagni, there are also the coordinates of the collecting locality.

## Geographic coverage

### Description

**Figure 2.**
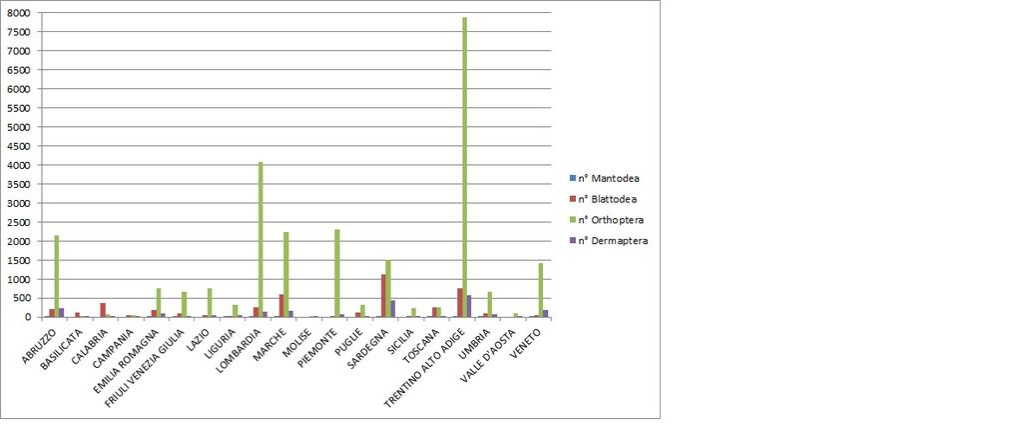
Number of samples collected on the Italian territory.

**Figure 3.**
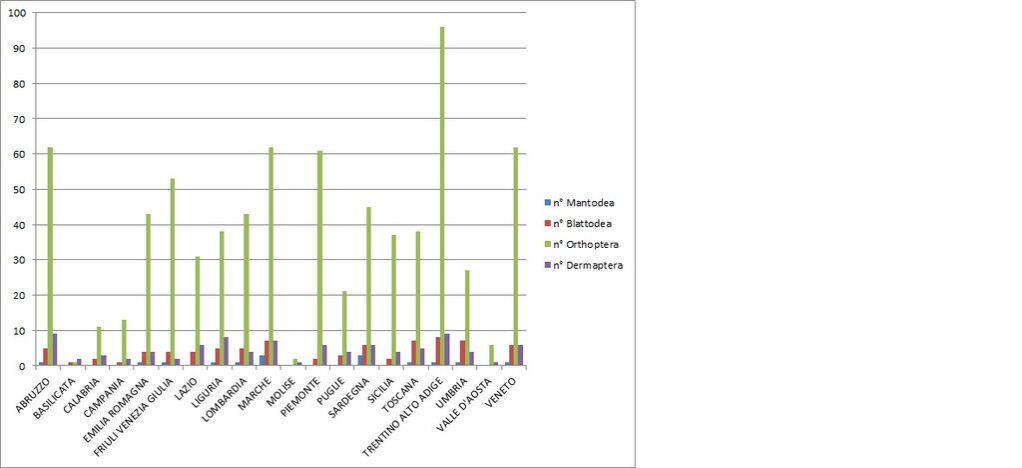
Number of species divided by Regions.

The Antonio Galvagni Collection is made up mostly (85%) of Italian specimens, plus others (15%) from the Mediterranean Basin and beyond. With this work, we want to underline the investigation carried out in the Italian regions. As shown in Table 1, Italian regions have different numbers of collected specimens and species: the Region Trentino Alto-Adige (TAA) is characterised by the higher number of collected specimens. This is due to the fact that Galvagni lived in TAA and, therefore, most of the excursions took place on his territory. This does not mean that some collections made in the other Regions are not complete; in fact, if the number of specimens by Regions are converted into the number of species for each Region, it is observed that most of the Regions in the systematic collection are optimally represented (Fig. 2, Fig. 3).

**Figure 4.**
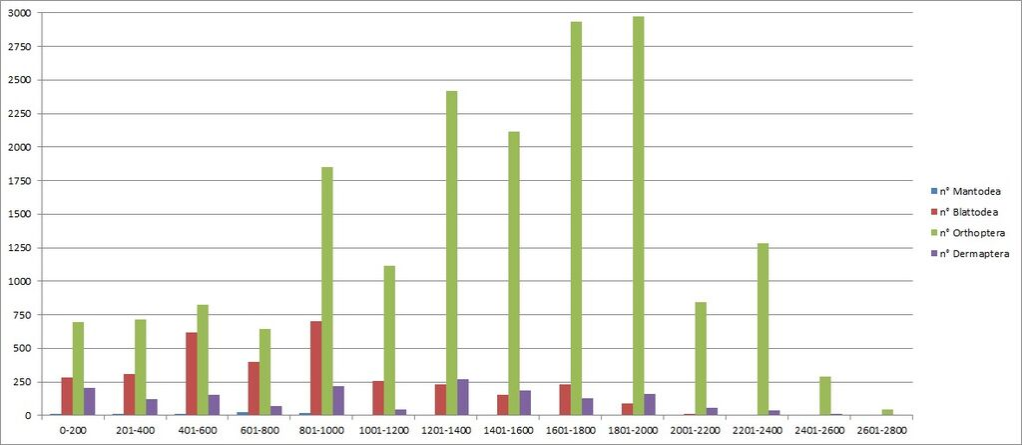
Number of samples collected at different altitudes.

Concerning the altitudinal distribution of Orthoptera, it is observed that there is a trend for which the Orthoptera increase with the altitude, showing a peak between 1800–2000 m, while the other orders are mostly found below 1400 m (Fig. 4). The reason for this is twofold: 1) a more intensive investigation effort on some medium-high mountain species, some examples are the genera: *Miramella* (n: 899), *Podisma* (n: 1438), *Anonconotus* (n: 521) and *Italopodisma* (n: 650);

2) in general, the number of specimens collected is lower on the valley floor or in coastal areas, as these areas are often highly anthropogenised, therefore lacking suitable habitats.

### Coordinates

 and Latitude; and Longitude.

## Taxonomic coverage

### Description

The 32,046 specimens of the Galvagni Collection consist of 138 Mantodea (5 species), 4,434 Blattodea (30 species), 25,014 Orthoptera (365 taxa), 2,450 Dermaptera (25 species) and 10 Phasmatodea (Table 1).

Although Antonio Galvagni collected all the orthopteroid groups, his studies concentrated on some genera and this is mostly evident by the fact that many specimens of target taxa are dissected and their genitalia prepared for a deeper study. Some examples of the most studied genera are: amongst Orthoptera *Rhacocleis* Fieber FX 1853 and *Pterolepis* Rambur 1838 with 205 samples, *Anonconotus* Camerano 1838 with 533 samples, *Miramella* Dovnar-Zapolskij 1932 with 898 samples, *Podisma* Berthold 1927 with 1460 samples and amongst Blattodea *Ectobius* Stephens, 1835 with 2966 samples.

In addition to these numbers, we also report a list of all those taxa described by Antonio Galvagni that are still valid for science (Fontana 2017). For the complete list of publications by A. Galvagni, see Fontana 2017.

### Taxa included

**Table:** 

Rank	Scientific Name	
order	Mantodea	
species	*Ameles andreae* (Galvagni, 1976)	
order	Blattodea	
species	*Ectobius caprai* Galvagni, 1971	
species	*Ectobius tamaninii* Galvagni, 1972	
species	*Ectobius tuscus* Galvagni, 1978	
order	Orthoptera	
subspecies	*Capraiuscola ebneri ebneri* (Galvagni, 1953)	
species	*Ephippiger ruffoi* Galvagni, 1955	
species	*Platycleis concii* Galvagni, 1959	
subspecies	*Metrioptera caprai baccettii* Galvagni, 1959	
species	*Rhacocleis baccettii* Galvagni, 1976	
species	*Rhacocleis bonfilsi* Galvagni, 1976	
species	*Pterolepis elymica* Galvagni & Massa, 1980	
subspecies	*Pterolepis spoliata kaltenbachi* Galvagni, 1981	
subspecies	*Pterolepis spoliata llorenteae* Galvagni, 1981	
subspecies	*Pterolepis spoliata nadigi* Galvagni, 1981	
subspecies	*Pterolepis spoliata nevadensis* Galvagni, 1981	
subspecies	*Pterolepis spoliata pascuali* Galvagni, 1981	
subspecies	*Pterolepis spoliata raggei* Galvagni, 1981	
subspecies	*Rhacocleis silviarum* Galvagni, 1984	
subspecies	*Pterolepis adolphorum* (Galvagni, 1988)	
subspecies	*Pterolepis claudiae* (Galvagni, 1988)	
subspecies	*Pterolepis moralesi* (Galvagni, 1988)	
subspecies	*Pterolepis berberica berberica* (Galvagni, 1989)	
subspecies	*Ctenodecticus bolivari africanus* Galvagni, 1990	
species	*Barbitistes vicetinus* Galvagni & Fontana, 1993	
species	*Pterolepis kabylica* (Galvagni & Fontana, 2000)	
species	*Pterolepis augustini* (Galvagni, 2001)	
species	*Anonconotus ligustinus* Galvagni, 2002	
species	*Anonconotus sibyllinus* Galvagni, 2002	
species	Dolichopoda (Dolichopoda) pavesii Galvagni, 2002	
species	*Anonconotus mercantouri* Galvagni & Fontana, 2003	
species	Dolichopoda (Dolichopoda) lycia (Galvagni, 2006)	
species	*Chrysochraon beybienkoi* Galvagni, 1968	
species	*Podisma magdalenae* Galvagni, 1971	
species	*Italopodisma lagrecai* (Galvagni, 1973)	
subspecies	*Heteracris adspersa massai* Galvagni, 1978	
genus	*Nadigella* Galvagni, 1986	
species	*Pseudopodisma transilvanica* Galvagni & Fontana, 1993	
species	*Pseudopodisma nagyi* Galvagni & Fontana, 1996	
order	Dermaptera	
species	*Chelidurella guentheri* Galvagni, 1994	
species	*Chelidurella vignai* Galvagni, 1994	
species	*Chelidurella fontanai* Galvagni, 1996	

## Temporal coverage

### Notes

**Figure 5.**
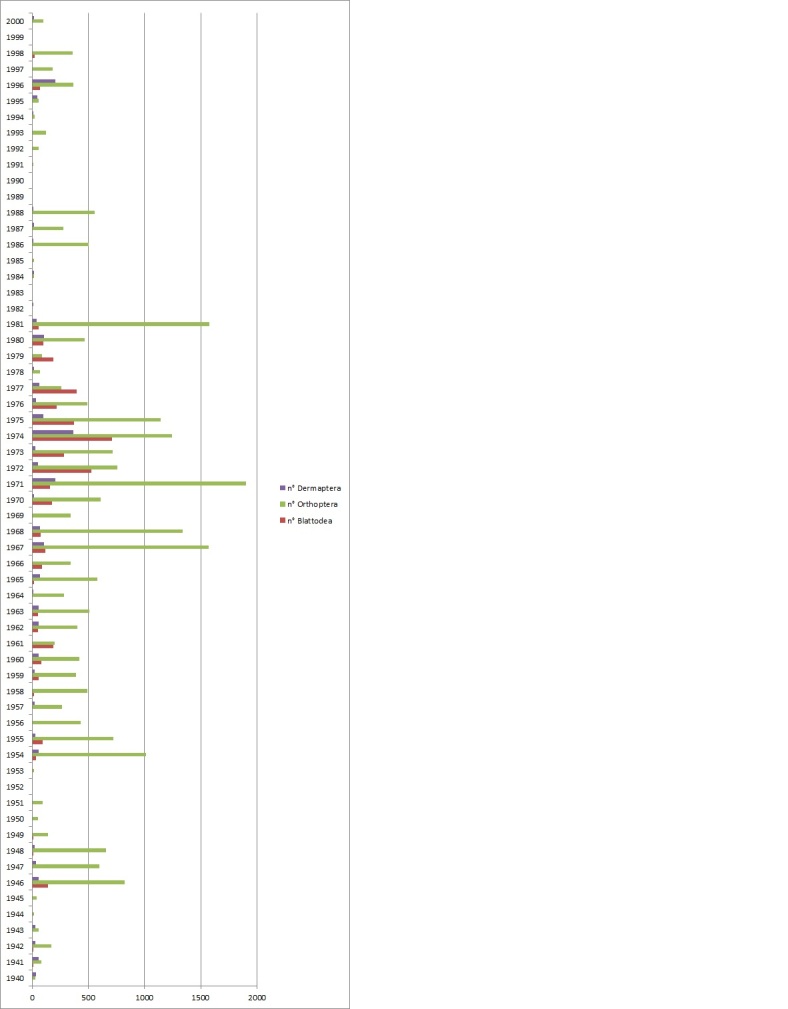
Number of specimens captured during the principal years of sampling.

Antonio Galvagni began to capture specimens intensively from 1940 and continued until he lost the strength to collect; his collection covers more than 60 years of Italian natural history. From the trend of the graph in Fig. 5 comes an alternation of periods of intense capture and periods of stasis. These years were probably used to study the previously accumulated material. These data are due not to the seasonal trend, but clearly to an alternation of collecting and study periods.

Even if there are peaks of collecting activity, during his whole life, Galvagni collected an average of 400 specimens every year.

## Usage licence

### Usage licence

Creative Commons Public Domain Waiver (CC-Zero)

## Data resources

### Data package title

Databasing of Antonio Galvagni Collection (Insecta: Blattodea, Dermaptera, Mantodea, Orthoptera)

### Resource link

https://www.fondazionemcr.it/extendedsearch_museo.jsp?id_schema=69&ID_LINK=113754&area=295&COL0047=&COL0052=&COL0053=&COL0054=&COL0055=&COL0056=&COL0058=&COL0062=Galvagni&COL0001=1&COL0002=1&COL0003=&COL0004=&COL0005=&COL0006=&COL0013=&COL0024=&btnSearch=Cerca

### Number of data sets

1

### Data set 1.

#### Data set name

collezione Galvagni

#### Number of columns

37

#### Description

The Galvagni Collection database can be downloaded as supplementary material (Suppl. material 1).

**Data set 1.** 

Column label	Column description
Section	Museum section to which the material is related
Sub-section	Museum Sub-section to which the material is related
Number of specimens	How many specimens are databased
Object name	Name of the species
Continent	Continent from which the specimen comes
Country	Country from which the specimen comes
Region	Region from which the specimen comes
Province	Province from which the specimen comes
City	City from which the specimen comes
Locality	Locality from which the specimen comes
Location	Institution where the specimen is preserved
Collection	Collection of the museum where the specimen is preserved
Specific Position	Number of the entomological box where the specimen is preserved
Board	Inventory paper board, when available
Phylum	Phylum to which the specimens belong
Class	Class to which the specimens belong
Order	Order to which the specimens belong
Family	Family to which the specimens belong
Genus	Genus to which the specimens belong
Species	Species to which the specimens belong
Species Author	Author of the species
Subspecies	Subspecies to which the specimens belong
Subspecies Author	Author of the subspecies
Number of males	Number of male specimens
Stage of males	Stage of development of the preserved specimens
Number of females	Number of female specimens
Stage of females	Stage of development of the preserved specimens
Mounting Method	Procedure used to prepare the specimens
Conservation status	Conditions of the specimens (good, bad, broken etc.)
Lowest altitude	Lowest altitude of the collecting locality
highest altitude	Highest altitude of the collecting locality
Collecting date 1	First date of period in which the specimens have been collected
Collecting date 2	Last date of period in which the specimens have been collected
Collector	Who collected the specimens
Reviewer	Who reviewed the data entry
Revision date	Date in which the revision was made
Notes	Additional info about identification, type material etc.

## Additional information

### Conclusion

Natural History Museums collections are important for homeland security, public health and safety, monitoring of environmental change, taxonomy and systematics (Suarez and Tsutsui 2004). More specifically, entomology collections serve, amongst others, for pest identification, past and present biodiversity assessment, public education, conservation and recovery of endangered species (https://www.entsoc.org/sites/default/files/files/Science-Policy/ESA-PolicyStatement-EntomologicalCollections.pdf). The last of this topic is particularly important in recent research run by staff of the Zoology Section of FMCR, since the specimens in recently-acquired collections of Orthoptera (Galvagni collection and Fontana Collection) have been essential in inter-institutional projects about two interesting species, i.e. *Uromenus annae* (Targioni-Tozzetti, 1881) from Sardinia and *Zeuneriana marmorata* (Fieber, 1853): the specimens preserved in the Collections have been on the basis of the correct identification of newly-discovered populations for both species (Buzzetti et al. 2019).

The Italian Natural History Museums are in a difficult situation due to many factors (Andreone et al. 2014) and the main dangers for entomology collections are staff and fund reductions plus insufficient training and expertise. Collections themselves can be a useful tool against the last of this threat as specialists can focus on museum material and can gather in museum institutions to share knowledge and train young researchers. As the public and managers become aware of the importance of historic entomology collections, we strongly encourage administrators and students to evaluate possible solutions and careers on entomology collections, in a modern way to take care of the environment.

## Supplementary Material

00A290A7-4B92-5558-A673-1BC5DEB4E496 10.3897/BDJ.9.e65953.suppl1 Supplementary material 1 Collezione Galvagni Data type Geographic distribution, number of specimens, sex, collecting date, notes File: oo_519306.txt https://binary.pensoft.net/file/519306 Fondazione Museo Civico di Rovereto

## Figures and Tables

**Table 1.** Total of specimens, types and holotypes conserved in the Galvani’s systematic collection.

Orders	Number of specimens	Types	Holotypes
Mantodea	138	1	1
Blattodea	4434	32	3
Orthoptera	25014	320	33
Dermaptera	2450	29	3
Phasmatodea	10	0	0
